# EOG-sEMG Human Interface for Communication

**DOI:** 10.1155/2016/7354082

**Published:** 2016-06-21

**Authors:** Hiroki Tamura, Mingmin Yan, Keiko Sakurai, Koichi Tanno

**Affiliations:** ^1^Department of Environmental Robotics, University of Miyazaki, Miyazaki 889-2192, Japan; ^2^Organization for Promotion of “Center of Community” Program, University of Miyazaki, Miyazaki 889-2192, Japan; ^3^Interdisciplinary Graduate School of Agriculture and Engineering, University of Miyazaki, Miyazaki 889-2192, Japan; ^4^Department of Electrical and Systems Engineering, University of Miyazaki, Miyazaki 889-2192, Japan

## Abstract

The aim of this study is to present electrooculogram (EOG) and surface electromyogram (sEMG) signals that can be used as a human-computer interface. Establishing an efficient alternative channel for communication without overt speech and hand movements is important for increasing the quality of life for patients suffering from amyotrophic lateral sclerosis, muscular dystrophy, or other illnesses. In this paper, we propose an EOG-sEMG human-computer interface system for communication using both cross-channels and parallel lines channels on the face with the same electrodes. This system could record EOG and sEMG signals as “dual-modality” for pattern recognition simultaneously. Although as much as 4 patterns could be recognized, dealing with the state of the patients, we only choose two classes (left and right motion) of EOG and two classes (left blink and right blink) of sEMG which are easily to be realized for simulation and monitoring task. From the simulation results, our system achieved four-pattern classification with an accuracy of 95.1%.

## 1. Introduction

During eye movements, a potential exists across the cornea and the retina, and it is the basis for the electrooculogram (EOG). The EOG can be modeled by a dipole [1] and used in medical systems. Several EOG-based human-computer interface studies are found in the literature. For example, a wheelchair controlled with eye movements is being developed for disabled and elderly people. The eye movement signals and the sensor signals are combined, and both direction and acceleration are controlled [2]. Surface electromyogram signals (sEMGs) are detected over the skin surface and are generated by the electrical activity of muscle fibers during contraction [3]. Moving muscles can be detected by analyzing sEMGs. One of the important applications of sEMGs is controlling artificial legs. Although head movement, which is a natural gesture, can be used to indicate a certain direction [4], seriously disabled people cannot move their neck and head. However, many of these people can employ facial muscle movement. sEMG is a way of studying facial muscle activities by recording the action potentials from contracting fibers. sEMG can be detected with surface electrodes, which are easy to apply and noninvasive and pose no health and safety risks to the users. Computer systems can also be controlled by using face sEMG signals [5, 6]. These computer systems can recognize facial motion (left blink, right blink, and bite) by using sEMG sensors. Furthermore, EOG, electroencephalogram (EEG), and EMG signals can be classified in real time and can control movable robots by using an artificial neural network classifier [7].

Investigating the possibility of employing EOGs for a human-computer interface, the relation between the sight angle and an EOG is determined. In-depth studies have found that the slowly changing baseline drift makes it difficult to estimate continuous EOG signals, and this drift only appears in direct current (DC) signals in the circuit. To overcome this issue, our system previously proposed the use of alternating current (AC) of EOGs to reduce the baseline drift by segmentation of the signal [8–10]. In these papers [8–10], we introduced the mouse cursor control system using our EOGs technique. The initial thresholds of the eyes movement class (right, left, and voluntary blink) are empirically determined individually for each user. Two eyes movement classes (right and left) are command of the same movement of the mouse cursor. In these papers [8–10], the eyes movement of diagonally lower right direction is a command of click processing. In addition, the voluntary blink is a command of click processing, too. However, these systems have a problem in which the face sEMG signals become noisy for EOG signals.

In this study, we are developing an EOG-sEMG human interface system for communication. Our proposed EOG device does not have the problem of artifacts from eye blinking. We apply an algorithm that uses the both dynamic variation of the DC element and the pattern classification of the AC element. This segmentation of the signal reduces baseline drift. Although there was a 3-electrode method which could measure vertical and horizontal components in EOG which was considered as the noise during the EEG measuring [11], our EOG system still uses cross-channels which use 4 electrodes to both improve the accuracy of EOG measuring and realize the EOG-sEMG “dual-modality” process simultaneously. Furthermore, the cross-channel EOG signals have a similar performance to the plus-channel method which is widely used in EOG measuring. In addition, the electrode position of cross-channel EOG signals method has a good feature that sEMG can also be effectively measured at the same time. In comparison with other papers [12], this is a useful merit.

Compared with other related researches on human-computer interfaces for helping people with disabilities such as eye tracking systems [13], which use image processing, the EOG-sEMG based on our proposal human-computer interface has strong anti-interference ability from the environment such as lights and noises. The patients could use this type of interface even without sight. In particular, the ALS patients could still control their eyeballs and muscles around the eyes even in terminal stage, in which they could use the EOG-sEMG based on our proposal human-computer interface also. Although image processing devices are widely used because of being intuitive and more predictable, the EOG-sEMG based on our proposal human-computer interface still provides good choice for the severely handicapped persons.

In this paper, we propose a technique that can perform face pattern recognition by recording the EOGs and sEMGs. In order to prove the performance of our proposed method, we tried 3 related experiments step by step. At first, although as much as 7 classes could be recognized, dealing with the real state of the patients and the initial accuracy tests, we only choose two classes (left and right motion) of EOG and two classes (left blink and right blink) of sEMG which are easily to be realized for simulation and monitoring task. Next, we carried out experiments of character inputs by using our proposed system with those 4 classes. The experimental participants were healthy men in their twenties who agree to join in our experiments with no coercion. From these experiments, we showed that the four-pattern recognition of our proposed system is easy to learn and to use. Further, we applied our proposed simplified method (software that can do character input with one click (one pattern recognition)) for the severely handicapped persons (muscular dystrophy patients).

## 2. The Measurement System

### 2.1. Cross-Channels

**Figure 1:**
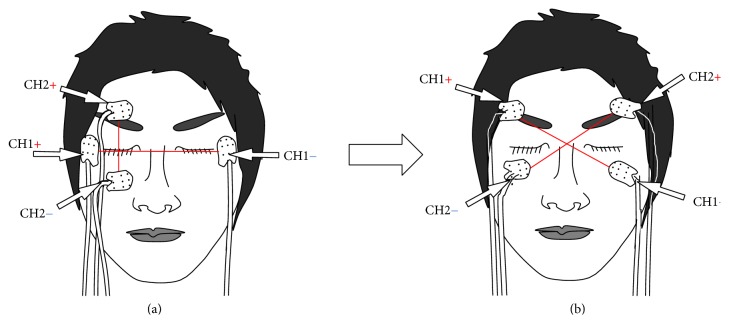
Conventional method (a) and the cross-channel method of the EOG (b) [8–10].

As shown in Figure 1(a), the plus-channel method is widely used as the most conventional way for recording EOG signals in the previous research: the horizontal signals were recorded by Channel 1 (CH1), and the vertical signals were recorded by Channel 2 (CH2) [8–10]. In this paper, to improve the accuracy of the EOG signals, a new cross-channel method is proposed as shown in Figure 1(b). The horizontal and the vertical signals could be recorded by both channels at the same time. It is much easier to analyze data using double signals.

### 2.2. The EOG-sEMG Measurement System

**Figure 2:**
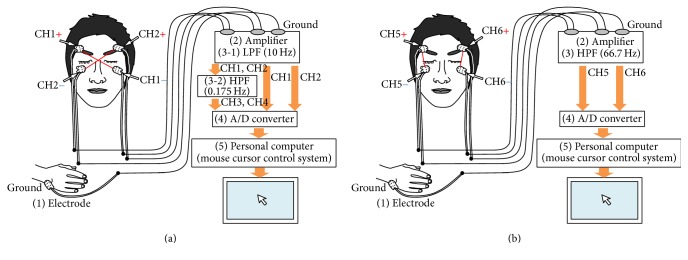
Proposed EOG-sEMG human interface system: (a) EOG flow and (b) sEMG flow. The EOG flow and the sEMG flow use the same five electrodes.

In this subsection, the design of the EOG-EMG measurement system is proposed.  Figure 2 shows the formal scheme for acquisition and analysis of the EOG and sEMG signals for control and flow of information through the system. Our proposed system has four components: (1) amplifier, (2) filter, (3) A/D converter, and (4) mouse cursor control system. Specifically, this means the system consists of five electrodes, an A/D converter, a personal computer, and a monitor (shown in Figure 2). Four electrodes for the two channels of sEMG signals are pasted on the face, and one electrode is pasted on the right or left wrist as the ground.

### 2.3. The EOG-sEMG Sensing Device

**Figure 3:**
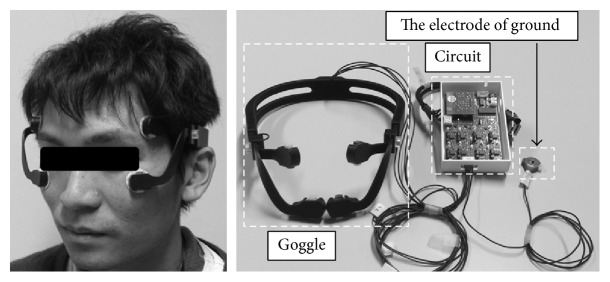
Interface device to fix the electrodes on the face.

**Figure 4:**
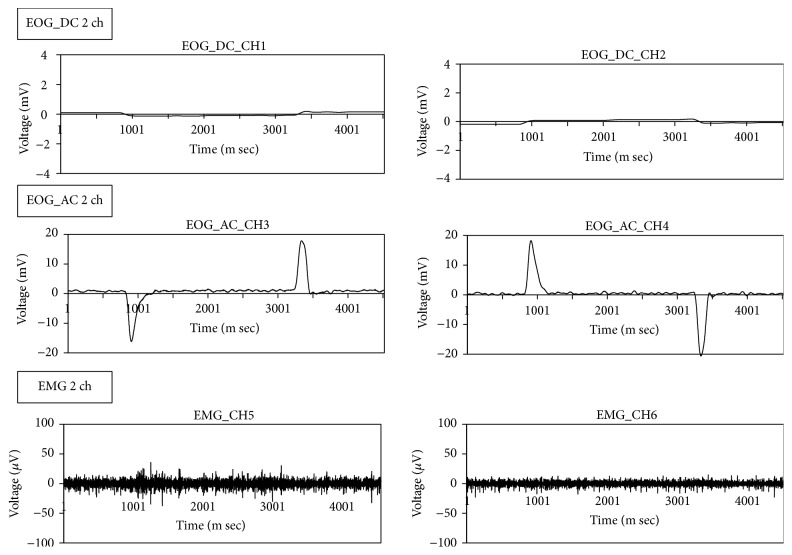
Data sent from the interface device. The signals are sEMG 2 ch and EOG 2 ch. The EOGs measure the AC and DC signals.

In this subsection, we show the interface device used in this study.  Figure 3 shows a person wearing the interface device. This interface device resembles goggles or glasses, fixing the electrodes. The signals measured by the device are sent to the PC using Bluetooth. The sent data are shown in Figure 4. The measured signals are DC currents for two EOG channels (2 ch), which are CH1 and CH2, and AC currents for two EOG channels (CH3 and CH4). In the same time, 2 ch signals of sEMG are also measured, which are CH5 and CH6. We will show the details of the measured data in the following section.

## 3. The EOG Recording System

**Figure 5:**
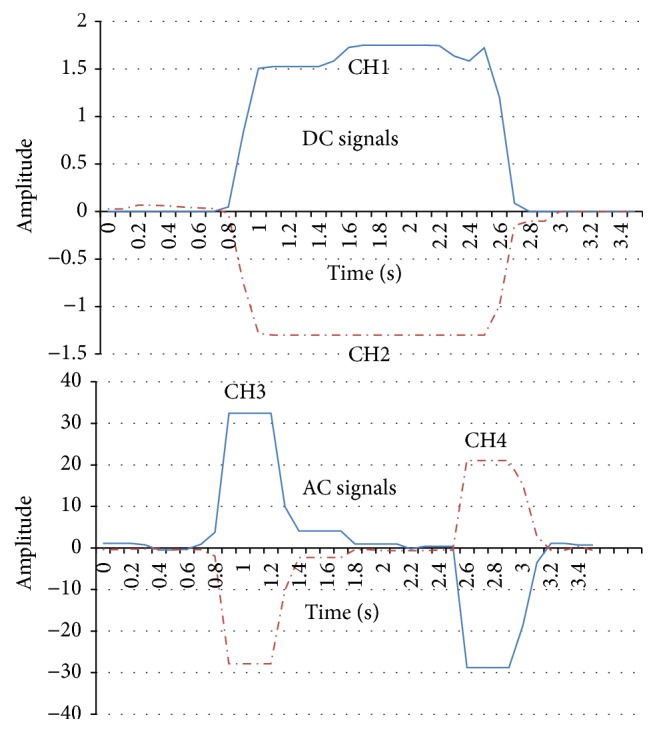
The EOG signals recording samples in CH1, CH2, CH3, and CH4 (right).

**Figure 6 fig6:**
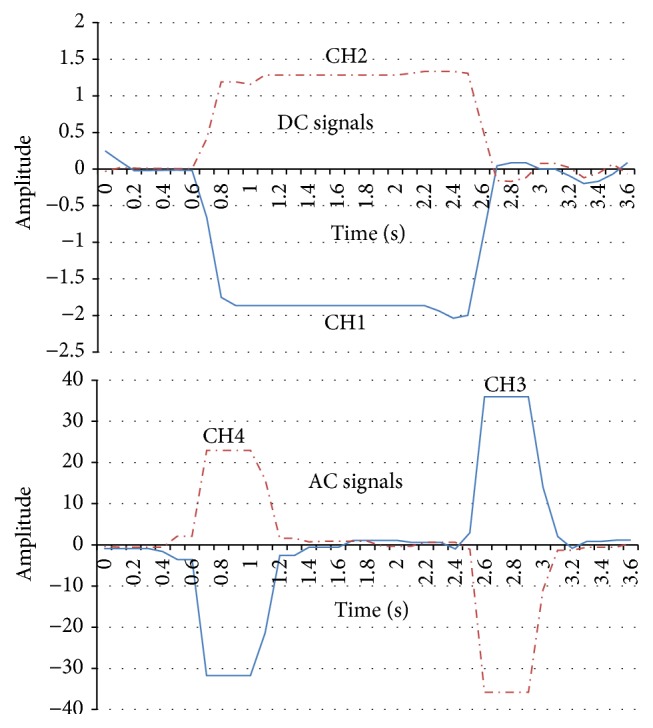
EOG signal recording samples in CH1, CH2, CH3, and CH4 (left).

**Figure 7 fig7:**
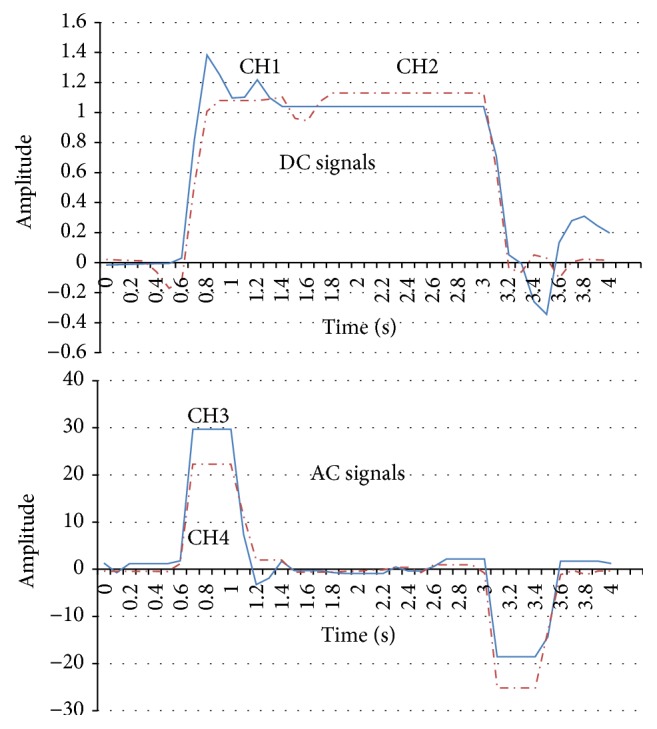
EOG signal recording samples in CH1, CH2, CH3, and CH4 (up).

**Figure 8:**
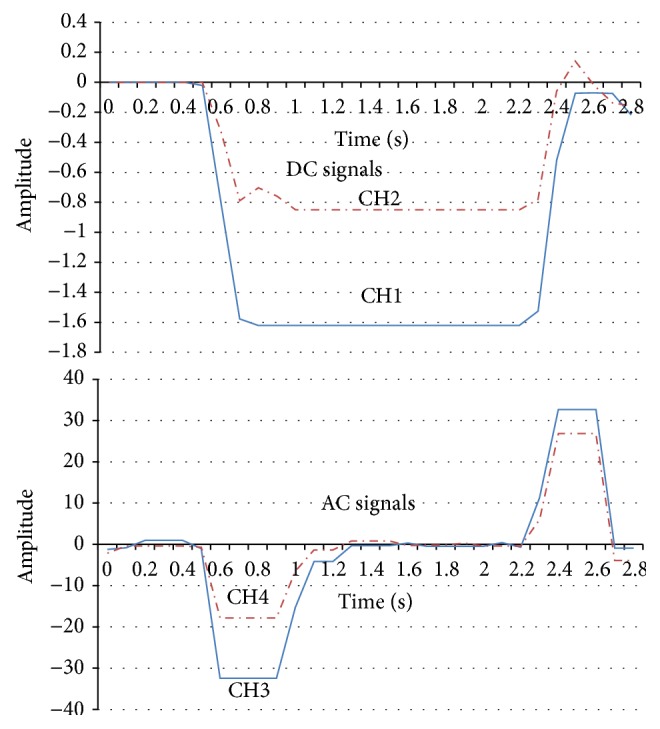
EOG signal recording samples in CH1, CH2, CH3, and CH4 (down).

In this section, we will introduce the EOG recording system. Our proposed EOG system is based on two flows: (1) the amplifier and the low-pass filter (DC element) and (2) the amplifier, low-pass filter, and the high-pass filter (AC element). After recording the signal amplitude (1000 times) and noise reduction measures for the biopotential data acquisition system, four kinds of eye movements (right, left, up, and down) are recognized accurately and the electronic noise reduction is also successful. It should be noted that the horizontal EOG signals are stronger than the vertical EOG signals. That is because vertical saccades are slower than horizontal saccades, and downward saccades are the slowest [14]. Figures 5 [Fig fig6] [Fig fig7]–8 show that the four eye movements (right, left, up, and down) are clearly different. Moreover, CH1 and CH2 are DC signals, which can be used for recording the continuous movements of the eyes. CH3 and CH4 are AC signals of the EOG. Therefore, CH3 and CH4 strongly react to eye movements only.

**Figure 9:**
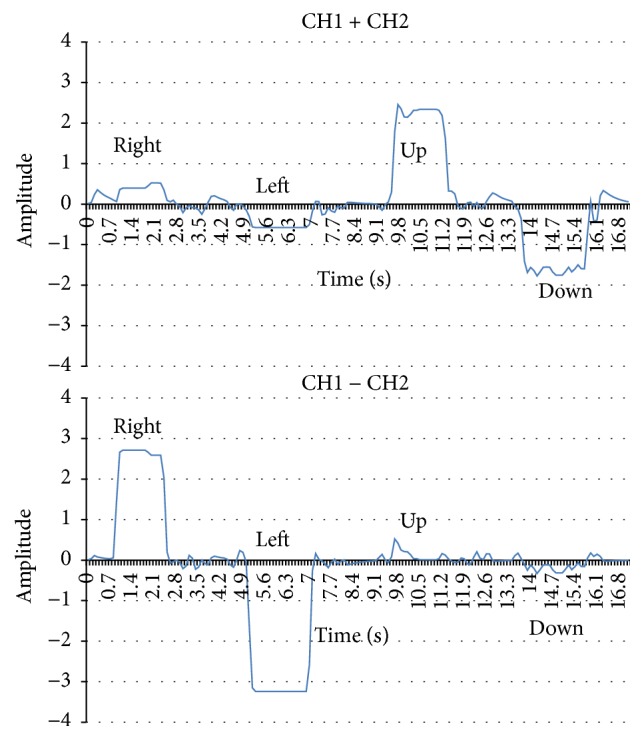
EOG signal recording samples in CH1 + CH2 (upside) and CH1 − CH2 (downside).

Because the EOG signals change for all four eye movements, we imported CH1 + CH2, which is used for the vertical signals and CH1 − CH2 for the horizontal signals. In the experiments, we asked each participant to move his eyeballs to follow the next sequence: center-right-center-left-center-up-center-down-center. The results of these two new procedures are shown in Figure 9.

## 4. sEMG Signal Recording

Figure 2 shows the formal scheme for the acquisition and analysis of the sEMG signals for control and flow of information through the system. Our system consists of these four components: (1) surface electrodes, (2) amplifier, (3) high-pass filter, and (4) personal computer for sEMG signal classification. The sEMG signals detected by the surface electrodes are amplified and filtered prior to data acquisition to reduce noise artifacts and enhance spectral components that contain the information for data analysis. Two channels of sEMG signals can be used to recognize facial movement. To remove the DC level and the noise of the 60 Hz power line, the high-pass filter is used. The cutoff frequency of the high-pass filter is 66.7 Hz.

**Figure 10:**
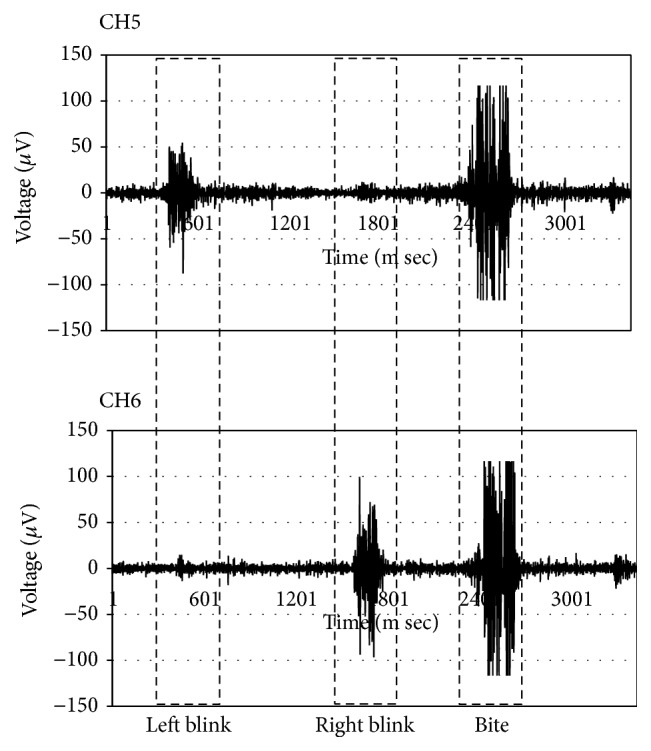
Activation levels of two-channel sEMG.

The recordings in Figure 10 show the applied noise reduction measures in our system. As a result, the data of the three sEMG classes (right blink, left blink, and bite) are clearly different. After filtering and amplifying (approximately 1000 times), the sEMG signals are digitized and then transferred to the personal computer. The sampling frequency of the measurement data is 1 KHz on a band from 0 Hz to 500 Hz.

**Figure 11:**
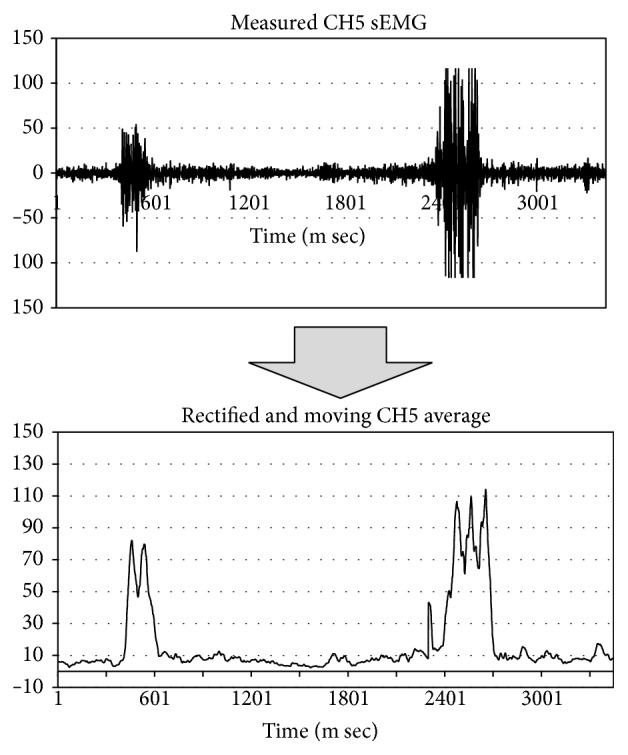
Diagram of process. SEMG signals are computed to obtain the rectified and moving averages. The signal is determined to be active or inactive based on the threshold.

The sEMG signals are processed by the moving average processing. The moving average processing calculates the rectified and unweighted mean of the previous *n*  (*n* = 50) data points. Then, the value after the moving average processing is determined as “active” or “inactive” based on the threshold. The thresholds (CH5 and CH6) are set according to the users. This method is necessary to set the threshold value of each user. Moreover, this system does not react to the usual blink.  Figure 11 shows a diagram of this process.

## 5. Pattern Recognition Algorithm

**Figure 12:**
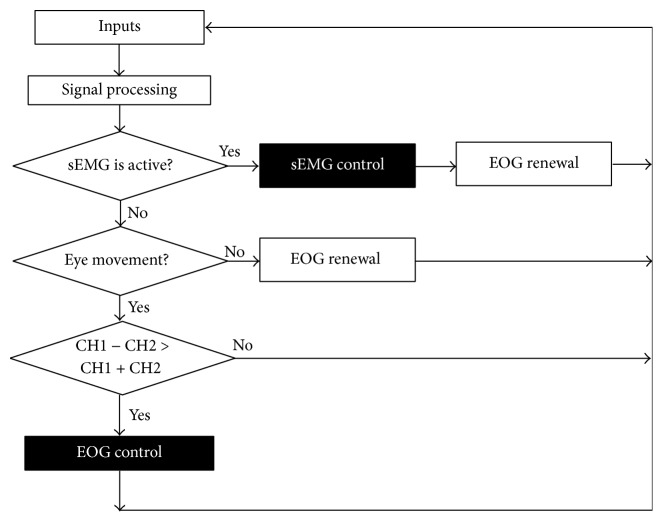
Flow of EOG-sEMG pattern recognition algorithm.

In this section, we will introduce our proposed EOG-sEMG pattern recognition algorithm.  Figure 12 shows the overall flow of our proposed system. This process consists of repeating steps. The pattern recognition consists of five classes: two classes (left and right movement) for the EOG and the three classes (left blink, right blink, and bite) for the sEMG.

If the sEMG data after signal processing exceeds a threshold, the pattern of the sEMG data is determined by CH1 only (right blink-like motion) or CH2 only (left blink-like motion) or both (the bite or strong blink). Our proposed algorithm initializes the DC elements of the EOG after the sEMG activity is completed. Furthermore, when the AC element of the EOG has not changed (eyes are not moving) and the sEMG is not active, our proposed algorithm initializes the reference value which eyes are looking as the front (“EOG renewal” in Figure 12). In other words, our system determines that eyes are looking at the front, because AC element is not changed. At that time, our system updates as the reference value of the DC elements. After that, our system uses the amount of change in DC from this reference value.

From the experimental rule, judging threshold between active and inactive is set. Next, when the eyes move, our algorithm compares the changing range of CH1 + CH2 and CH1 − CH2. When CH1 − CH2 is larger, our algorithm performs the determination process of the EOG. When CH1 + CH2 is larger, our algorithm determines that the eyes moved in the vertical direction, because the EOG data for the vertical direction of the eyes is similar to the EOG data for the blink pattern. In addition, our experimental results indicate that many people cannot easily control the up direction of the eye.

**Figure 13:**
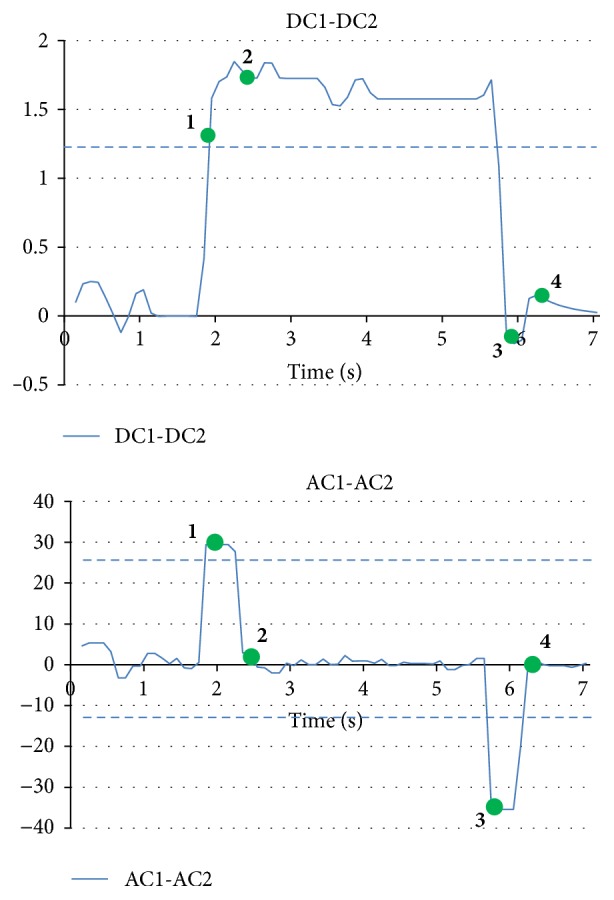
Example of EOG pattern recognition processing. The bold numbers represent the algorithm steps.

Next, we introduce the EOG pattern recognition algorithm, an example of EOG pattern recognition processing shown in Figure 13. Although Figure 9 shows that all of eyes motions could by checking values for CH1 + CH2 and CH1 − CH2, pattern marching algorithm is still necessary to solve the baseline shift caused by drift problem. The algorithm steps of Figure 13 are as follows:

(**1**) The DC and AC elements of the EOG exceed the threshold for the right direction. It is determined that this is the right direction of the eyes.
(**2**) The DC element continues to exceed the threshold for the right direction and the AC element returns to the baseline. The direction of the eyes continues to be to the right.
(**3**) The AC element greatly changes in the negative direction when the eye direction returns to the center position.
(**4**) The AC element and the DC element return to each element's baseline. Then, the baseline is updated.

Our system performs the determination of the right and left movement of the eyes by using this algorithm. This algorithm makes it possible to determine direction. This is a difficult process when using only the AC element.

## 6. Experiments and Results

To test the effectiveness of our proposed system, we conducted two experiments: one is a pattern recognition test and the other is a character input test.

### 6.1. Pattern Recognition Experiment

**Figure 14:**
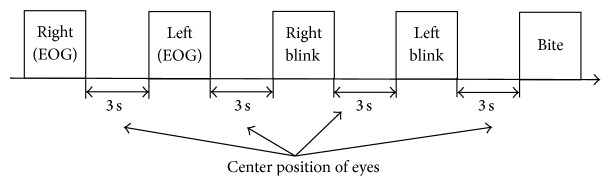
Basic tasks in pattern recognition experiment.

First, we conducted pattern classification experiments for the two eye movement classes (right and left) and the three facial sEMG classes (right blink, left blink, and bite). The interval of each action is 3 seconds (shown in Figure 14). Each participant performed each eye movement nine times. In addition, we tested whether our proposed system could reject a normal blink. We carried out the normal blink rejection test 30 times. The participants of the experiments were eight healthy males who are all the numbers of our lab in their twenties who gave their consent to participate in this experiment.

### 6.2. Pattern Recognition Experimental Results

The pattern recognition experimental results are shown in Table 1 and the last line shows the performance evaluation (PE) of each pattern under 5 points rule (correct for 5 points, reject for 2 points, and miss for 0 points). Reject means that input pattern did not react. Miss means that input pattern was wrong response. From the experiments, the EOG pattern recognition (right and left) of our proposed system is reliable. From these experimental results, our proposed system shows good performance in the recognition of the four classes (right, left, right blink, and left blink). The average recognition rate was 95.1%, the average reject rate was 1.4%, and the average miss rate was only 3.5%, and the average PE is 4.79 of 5.

However, the sEMG bite pattern recognition is not as good as expected. One reason is that some people have troubles of occlusal irregularity that cause divergence between their cheeks. This kind of bite actions was usually recognized as right or left blink. Another reason is that we used a threshold tuning method for distinguishing normal blink, and the normal blink rejection test had a 97% success rate (232/240). Under this method, the bite actions which could not pass the threshold will be rejected the same as the normal blink.

### 6.3. Character Input Experiment

**Figure 15:**
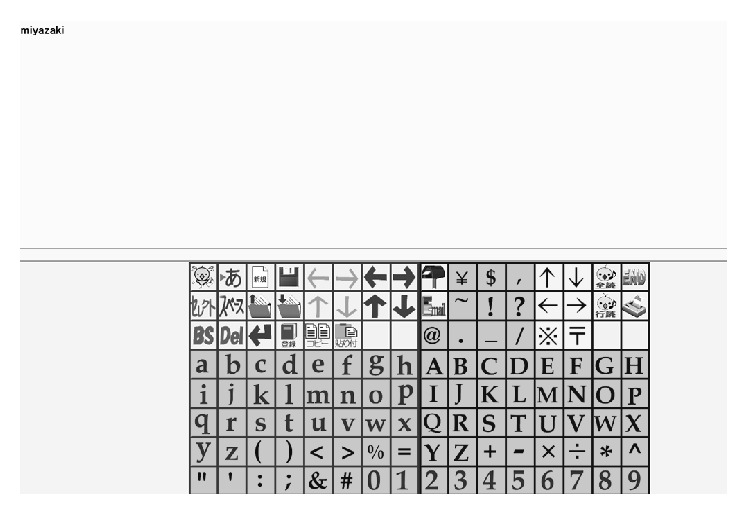
The alphabet sentence input software for the “miyazaki” task.

Next, we conducted the character input experiment using the four classes (right (EOG), left (EOG), right blink (sEMG), and left blink (sEMG)). The alphabet sentence input software, Hearty Ladder [6], is shown in Figure 15. We used the four-division selection method. We assigned the four patterns as follows:

 Right (EOG) is the command to select lower right area.
 Left (EOG) is the command to select lower left area.
 Right blink (sEMG) is the command to select higher right area.
 Left blink (sEMG) is the command to select higher left area.

The user repeated the filtering of four patterns until the character of the last pattern was selected. The control interval was 0.1 s. We conducted experiments of the “miyazaki” input task (8 characters: 4 operations for 1 character) which is the name of our university. The participants of the experiments were nine healthy males in their twenties who gave their consent to participate in this experiment. The experimental participants included six people experienced with this system.

### 6.4. Character Input Experimental Results

We tried five trials of the “miyazaki” input task. We counted the number of misses and the time duration until the end of input. The experimental results are shown in Table 2. From these experiments, the average time of one trial was 50.0 s. The average input time of one character of the alphabet was approximately 6.3 s (50 s/8 characters) in our proposed system. The time of the fastest participant, K.M, was 4.5 s (35.8 s/8 characters). In addition, if we considered the participants M.F, M.S, and R.K who had been using our proposed system over 100 h as the experienced and the other 6 participants who did not use our proposed system by any chance as inexperienced, the average time of the experienced participants was 49.2 s and that of the inexperienced participants was 51.5 s. The miss rate of the inexperienced participants was also low. From these results, we consider that one advantage of our proposed system is that users do not need a lot of training.

We compared the performance of our proposed method with the EOG method and the sEMG method. The EOG method used right and left eye motions only as the 2-division selection method. The sEMG method used right blinks and left blinks only as the 2-division selection method. We tried the same experiments of five trials of the “miyazaki” input task with the same participants. These experimental results are shown in Table 3. It can be seen that our proposed method has the best performance of all the three methods. We calculated the time required for one operation. The EOG method was 1.93 s, the sEMG method was 1.65 s, and our proposed method was 1.56 s. In addition, the miss rate of the sEMG method was less than that of our proposed method. One of the most conclusive reasons is that the number of operations (7 operations for 1 character) is bigger in the 2-division selection method than the 4-division selection method.

As we mentioned in this section, 2 or 4 patterns could be recognized by our proposed system. All these patterns could be assigned as separated functions and, dealing with the state of users, all these patterns could be employed by any combination of them. In the next section, we will discuss the application of our proposed system for the severely handicapped persons using only 1 pattern.

## 7. Experimental Results of the Severely Handicapped Persons

In this paper, we applied our proposed method for the severely handicapped persons (muscular dystrophy patients). We have gotten the approval of the ethics committee of the University of Miyazaki. The experiment of Section 6 with complicated operation will cause great fatigue to the people with disabilities, so experiments were carried out in the way that one character will be entered with a single click using EOG or sEMG. Only one pattern was selected from EOG or sEMG patterns that could be easily used. In this character input method, character is automatically selected by software; user can be selected by clicking when the target character has been chosen by the software. In this experiment, clicking processing is carried out by the recognition of EOG or sEMG activity.

This experiment had the two subjects. In order to minimize the influences of physical condition of subjects and environments, we tried the same experiments 10 times on different occasions in different days. The continuous 30 minutes in one experiment was used for testing the Japanese character input.  Table 4 shows the experimental results. From Table 4, recognition rate of sEMG method is 99.2% and recognition rate of EOG method is 98.0%. The input time was slow about 1 to 2 seconds compared to healthy subjects. Therefore, the handicapped persons have the same result as healthy ones using a single click. From these results, we can say that our approach is effective in severely handicapped persons.

## 8. Conclusions

In this study, we introduced an EOG-sEMG human-computer interface device designed for patients suffering from amyotrophic lateral sclerosis or other illnesses. We use both cross-channels and parallel lines channels on the face with the same 4 electrodes. This system could record EOG and sEMG signals as dual-threading for pattern recognition simultaneously. Furthermore, our proposed method using the combination of AC and DC elements of EOG reduces the corresponding drift and enables continuous operation in the recording of eye movements. The experimental results showed that our proposed method is effective for four-pattern recognition (right (EOG), left (EOG), right blink (sEMG), and left blink (sEMG)). In particular, our proposed method demonstrated good performance for the character input experiments. The miss rate was only 1.4%. In addition, the results of inexperienced and experienced participants showed very little difference. From these results, we think that one advantage of our proposed system is that users do not need a lot of training.

Furthermore, we compared our proposed method with the EOG method and the sEMG method. Our proposed method required the shortest time for character input. Our proposed human-computer interface can be applied in the EOG system, the sEMG system, and the EOG-sEMG system. It is possible to use our system for patients who can only control their eye motions. It is also possible to use only the sEMG signals with a high success rate until the user is comfortable using the device. Our proposed human-computer interface has the advantage that it can be used depending on the situation.

We hope to be able to communicate with patients suffering from amyotrophic lateral sclerosis or other illnesses by using our system. In our future work, we plan to test many subjects and more severely disabled people.

## Figures and Tables

**Table 1:** Pattern recognition experimental results.

	Right	Left	Right blink	Left blink	Bite
K.G	8/9 (reject: 1)	9/9	9/9	9/9	4/9 (reject: 5)
T.N	9/9	9/9	9/9	7/9	8/9 (reject: 1)
M.Y	9/9	8/9	9/9	8/9	4/9 (reject: 5)
K.N	9/9	9/9	9/9	9/9	8/9 (reject: 1)
M.F	9/9	9/9	9/9	9/9	3/9 (reject: 6)
R.K	8/9	9/9	7/9	7/9	9/9
K.M	7/9 (reject: 2)	8/9 (reject: 1)	8/9	9/9	5/9 (reject: 4)
T.T	9/9	9/9	9/9	9/9	7/9 (reject: 2)

Ave.	*94%* (reject: 4%)	*97%* (reject: 1%)	*96%*	*94%*	*67%* (reject: 33%)
P.E 5	*4.78*	*4.87*	*4.80*	*4.70*	*4.01*

**Table 2:** Character input experimental results.

	Average time (sec)	SD	Average miss rate (%)
K.I	42.0	3.1	0.0
K.M	35.8	5.0	0.0
R.K	60.2	12.4	3.5
T.T	53.2	13.0	2.5
K.G	61.0	15.5	5.0
T.N	43.2	2.7	0.0
M.F (inexperienced)	68.6	5.5	0.0
M.S (inexperienced)	36.6	5.2	2.0
R.K (inexperienced)	49.4	19.8	0.0

Ave.	*50.0*	—	*1.45*

**Table 3:** Comparison of experimental results of our proposed method with the EOG method and sEMG method.

	Average time (sec)	SD	Average miss rate (%)
EOG method (2-division)	77.0	8.7	2.06
sEMG method (2-division)	66.0	14.1	0.07
Our method (4-division)	50.0	11.6	1.45

**Table 4:** The experimental results of the two handicapped persons.

	Average time of one character input (sec) (healthy person)	Average miss rate (%)
EOG method (1-click)	16.1 (13.5)	2.0
sEMG method (1-click)	14.6 (13.5)	0.8
